# Immune Responses to an Oral Cholera Vaccine in Internally Displaced Persons in South Sudan

**DOI:** 10.1038/srep35742

**Published:** 2016-10-24

**Authors:** Anita S. Iyer, Malika Bouhenia, John Rumunu, Abdinasir Abubakar, Randon J. Gruninger, Jane Pita, Richard Lako Lino, Lul L. Deng, Joseph F. Wamala, Edward T. Ryan, Stephen Martin, Dominique Legros, Justin Lessler, David A. Sack, Francisco J. Luquero, Daniel T. Leung, Andrew S. Azman

**Affiliations:** 1 Division of Infectious Diseases, Department of Internal Medicine, University of Utah School of Medicine, Salt Lake City, Utah, USA; 2 World Health Organization, Juba, South Sudan; 3 Republic of South Sudan Ministry of Health, Juba, South Sudan; 4 Division of Infectious Diseases, Massachusetts General Hospital, Boston, MA, USA; 5 World Health Organization, Geneva, Switzerland; 6 Department of Epidemiology, Johns Hopkins University, Baltimore, MD, USA; 7 Department of International Health, Johns Hopkins University, Baltimore, MD, USA; 8 Epicentre, Paris, France; 9 Division of Microbiology and Immunology, Department of Pathology, University of Utah School of Medicine, Salt Lake City, Utah, USA

## Abstract

Despite recent large-scale cholera outbreaks, little is known about the immunogenicity of oral cholera vaccines (OCV) in African populations, particularly among those at highest cholera risk. During a 2015 preemptive OCV campaign among internally displaced persons in South Sudan, a year after a large cholera outbreak, we enrolled 37 young children (1–5 years old), 67 older children (6–17 years old) and 101 adults (≥18 years old), who received two doses of OCV (Shanchol) spaced approximately 3 weeks apart. Cholera-specific antibody responses were determined at days 0, 21 and 35 post-immunization. High baseline vibriocidal titers (>80) were observed in 21% of the participants, suggesting recent cholera exposure or vaccination. Among those with titers ≤80, 90% young children, 73% older children and 72% adults seroconverted (≥4 fold titer rise) after the 1^st^ OCV dose; with no additional seroconversion after the 2^nd^ dose. Post-vaccination immunological endpoints did not differ across age groups. Our results indicate Shanchol was immunogenic in this vulnerable population and that a single dose alone may be sufficient to achieve similar short-term immunological responses to the currently licensed two-dose regimen. While we found no evidence of differential response by age, further immunologic and epidemiologic studies are needed.

Reports of large cholera outbreaks outside of historically endemic regions have become increasingly common during recent years. In 2014, 42 countries across the globe reported a total of 190,549 cases of cholera, of which 55% were from Africa and 15% from the Americas^1^. In South Sudan, cholera outbreaks accounted for 6,421 cases including 167 deaths in 2014 and 1,818 cases including 47 deaths in 2015^2^,^3^. Recent outbreaks have rekindled interest in new cholera control tools, including oral cholera vaccines (OCV); however, evidence supporting the efficacy of OCV has largely been generated in South Asia, where historical cholera exposure patterns, co-circulation of other pathogens and general population health may differ significantly from those in Africa, potentially influencing vaccine response^4^,^5^. A number of studies have illustrated differences in oral vaccine immunogenicity and efficacy across different locations and populations, with differences attributed to ecological factors like population density, water and sanitation infrastructure and other host factors including co-infections, malnutrition, and breastfeeding (in infants)^6^,^7^,^8^,^9^. These studies emphasized the need for geographically- or subpopulation-targeted vaccine efficacy studies focused on groups most likely to be included in vaccination.

Since 2013, a global stockpile of OCV, managed by the International Coordinating Group, has served as the primary mechanism to procure rapidly and deploy vaccine to cholera outbreaks and areas at high risk of an outbreak^10^. At the time of this study, only one of the three WHO-prequalified killed whole cell vaccines, Shanchol (Shanta Biotechnic, Hyderabad, India), has been used in the stockpile. All three vaccines are licensed as a two dose regimen with at least 14-days between doses.

Studies of OCV outside historically endemic regions are limited. Field effectiveness studies of the two-dose OCV regimen have estimated high levels of protection against clinical cholera in Haiti, Tanzania and Guinea, though these estimates may in part be driven by ‘herd’ protection, masking important differences in direct protection between individuals^11^,^12^,^13^,^14^. These studies have provided some, though weak and not statistically significant, evidence that the first dose may be moderately protective and that young children may be less protected from the vaccine. Further insight into both of these observations, particularly in populations at high risk for cholera outbreaks, may have profound impacts on future vaccination strategies.

Immunogenicity studies may provide more insight on individual responses to the vaccine. One immunogenicity study in Haiti showed Shanchol to be immunogenic in all age groups ranging from young children to adults, though seroconversion was lower in young children^15^. In Haiti, as in previous studies from Bangladesh^16^, India^5^,^17^,^18^, and Ethiopia^19^, there were no significant differences in (vibriocidal) immune responses after the second dose compared to those after the first^19^. While vibriocidal antibodies have been shown to be a non-mechanistic correlate of protection and are the most commonly used marker in immunogenicity studies^20^, this complement-mediated response is mostly derived from IgM responses to *V. cholerae* lipopolysaccharide (LPS)^21^ and protective immunity against cholera is serogroup specific, with serogroups defined by the O-specific polysaccharide (OSP) portion of the LPS^22^. Thus, measurement of OSP-specific antibodies may provide further information regarding isotype-specific (especially class-switched) responses to vaccine.

Here, we present results from an immunogenicity study conducted within a population at high risk for cholera in Juba, South Sudan. We enrolled a subset of vaccinees (n = 205) from a 2015 OCV campaign in a camp of internally displaced persons to measure their immune responses to the vaccine. We use this data to estimate the proportion of vaccinees seroconverting after each dose, and explore different aspects of the participants’ baseline and post-vaccination immunologic markers of cholera exposure. In particular, we compare seroconversion proportions between children and adults, and compare the responses after one and two doses of OCV.

## Methods

### Study Setting

Cholera outbreaks occur frequently in South Sudan with at least 6 outbreaks reported since 2006^23^. At the end of 2013 more than 1 million people were internally displaced and resettled in formal and informal camps. Due to the poor living conditions and high perceived risk for cholera, over 162,577 inhabitants in camps throughout the country were vaccinated with OCV in February 2014, including some individuals in this study population^23^. Although an outbreak caused by *Vibrio cholerae* O1 Inaba was reported approximately 2 months after the start of vaccination activities, the vaccinated camps were largely spared, with only 6,269 medically attended, suspected, cholera cases^23^. An unpublished case-control study measuring vaccine effectiveness in these populations though underpowered, pointed towards lower effectiveness of Shanchol in children, a population perhaps in most need of protection^24^.

Continued violence around the country in 2014 and 2015 came with new population movements, and by April 2015, a survey in the UN House Protection of Civilian camp (PoC) revealed that only 17% of the residents reported to have been vaccinated with OCV the previous year^25^. The South Sudan Ministry of Health, with the support of the WHO, undertook another pre-emptive OCV campaign targeting all 28,520 residents of the UN House Protection of Civilian (PoC) camp who were over 1 year-old in June/July 2015.

### Sample Size

The sample size was based on detecting a significant difference in the proportion of young children who seroconverted compared to adults. Assuming that 91% of adults and 73% of young children would seroconvert (based on findings from Charles *et al.*^15^) and that 20% of those participating would dropout we estimated that 82 participants per age group would be needed in order to have 80% power to detect this difference at a 0.05 significance level. We initially intended to enroll 328 participants from four age groups; 12–23 months, 24–59 months, 60 months to 17 years and 18 years and above.

### Recruitment and Follow-up

**Figure 1:**
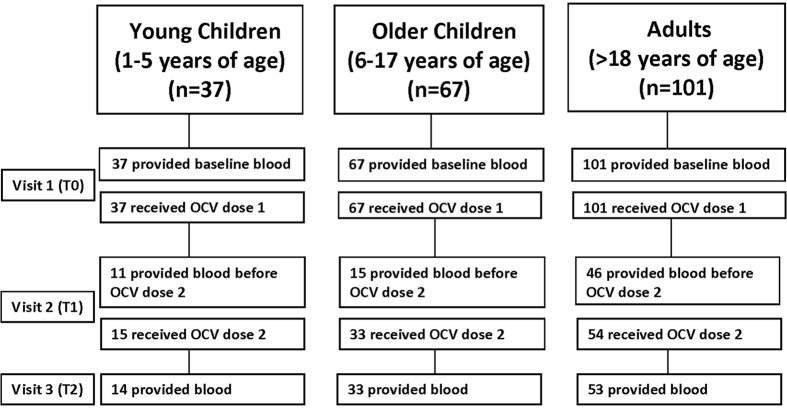
Enrollment and follow up of study participants (n = 205). Note that some participants did not provide blood at visit two but received the vaccine and then provided blood only at visits 1 and 3.

We recruited eligible individuals with no obvious or self-reported signs of acute gastrointestinal illness, residing in UN House PoC who reported plans to stay in the camp for at least 1 month (the planned duration of the study). Study staff recruited participants from vaccination posts, set-up throughout the camp, and through home visits starting 22-June-2015. Each participant provided written informed consent, answered a short questionnaire and provided a venous blood sample at the time of enrollment. In cases of participants between the age of 8 and 17 years, an assent was obtained in addition to the written, signed parental consent form. A physical examination was conducted for signs of malnutrition for children under five years old. Participants were asked to return to the study outposts 14-days later to provide another blood sample and to receive the second OCV dose. The final follow-up visit was scheduled 14-days after the second dose for the collection of blood (Fig. 1). Participants were reminded of their follow up visits either via phone calls or in person. Irrespective of their willingness to provide blood, a second dose of vaccine was offered.

### Sample Collection

Trained study nurses collected 3–5 mL of venous blood from each participant. The blood samples were transported in a cool box to a clinic within the camp (IMC clinic) or the National Public Health Laboratory, where lab technicians centrifuged the vials and placed the serum into two micro-tubes (cryovials). Serum sample were stored at −20 °C until the end of the study when they were then shipped on dry ice to the University of Utah for analyses.

### Vibriocidal Assays

Vibriocidal assays were performed as described previously^15^,^22^. Serially diluted heat inactivated test sera were incubated with target strains O1 Inaba (T19749) and Ogawa (X25049) and guinea pig complement (Sigma Aldrich, catalog # S1639). Titers were defined as the reciprocal of serum dilution that resulted in 50% or greater reduction in O.D. compared to serum free controls^15^,^22^. An internally generated, pooled serum sample served as the positive control and helped in accounting for inter-experimental variation. Samples were tested in duplicates. The threshold for inter- and intra-experimental variation was set at 2-fold. Seroconversion was defined as ≥4-fold rise in vibriocidal titers compared to baseline, following convention. Given the high inter-laboratory variation in vibriocidal titers, we standardized our vibriocidal protocol by comparison with de-identified serum samples with known vibriocidal titers obtained by a previous publication from Haiti^15^.

### OSP ELISAs

We used an OSP:BSA conjugate to measure OSP-specific antibody responses to the two O1 serotypes, Inaba and Ogawa as described previously^26^. Diluted test sera and positive control were added to Inaba- or Ogawa- specific OSP:BSA conjugate coated plates. Goat anti-human horseradish peroxidase conjugated IgG, IgM and IgA were used for detection. Plates were developed with O-phenylenediamine dihydrochloride substrate in the presence of H_2_O_2_ and read kinetically at 450 nm for 5 minutes. Samples were tested in triplicates. Samples were normalized with positive control (pooled sera) and are expressed as geometric mean ELISA units.

### Statistical Analyses

Vibriocidal antibody titers were log_2_ transformed for statistical tests. Within group comparisons were performed using Mann Whitney U-test. Analysis of Variance (ANOVA) with Kruskal-Wallis test was used for comparing results between groups. Fisher exact tests were used to compare proportion of seroconverters between groups. Spearman’s correlation test was used for assessing correlations. Two-tailed p-values of ≤0.05 were considered statistically significant. We applied the Bonferroni correction for multiple comparisons across age groups.

### Ethics Statement

All study staff, most of whom were recruited from the community, received theoretical and practical training about the study methodology and human subjects research ethics. The study protocol was reviewed and approved by the ethical review committee of the South Sudan Ministry of Health and the institutional review boards of Johns Hopkins Bloomberg School for Public Health and the University of Utah and carried out in accordance with relevant guidelines and regulations. Given that this study was embedded in a large public health campaign, and was not an intervention study, it was not registered in a WHO primary clinical trial registry.

### Data Availability

All data used in this manuscript are available at https://github.com/scottyaz/juba-ocv-immuno. Additional protocol details and raw data files are available from the corresponding author on reasonable request.

## Results

### Study enrollment and Participation

We initially aimed at enrolling a total of 328 participants stratified based on age groups; 12–23 months, 24–59 months, 60 months to 17 years and 18 years and above. However, since our enrollment was well under target, we modified our age groups, consistent with literature^15^. We enrolled 205 participants of which 37 (18%) were young children aged 1–5 years, 67 (33%) older children 6–17 years, and 101 (49%) adults ≥18 years. Volunteers provided blood and received dose 1 of OCV at the first visit (T0, day 0). The 2^nd^ visit was scheduled 14 days after the primary OCV dose; however, security and logistic issues delayed many of the visits leading to a median visit at 21 days after first dose (Interquartile range (IQR): 14–22 days; T1). During this visit (T1), only 30% of the young children (37), 22% of the older children (67) and 46% of the adults (101) enrolled provided blood. However, nearly all who received the second OCV dose at T1 provided blood at the third visit, T2 (median: day 35, IQR 29–36 days) (Fig. 1).

### Baseline characteristics of the study participants

Young children, older children and adults were stratified further, based on baseline (T0) vibriocidal titer cut-off of 80, a commonly used threshold to indicate recent exposure to cholera^15^. Age, sex distribution, proportions of individuals with history of OCV and cholera were similar within these age groups regardless of baseline vibriocidal titer (Table 1). The majority of the participants (85%) reported no diarrheal symptoms in the week before vaccination (Table 1). A significant proportion of individuals had a self-reported history of receiving an OCV in a vaccination campaign about one year earlier (90/205; 44%)^23^. Young children were evaluated further for their physical and nutritional status (Supplementary Table 1).

**Figure 2:**
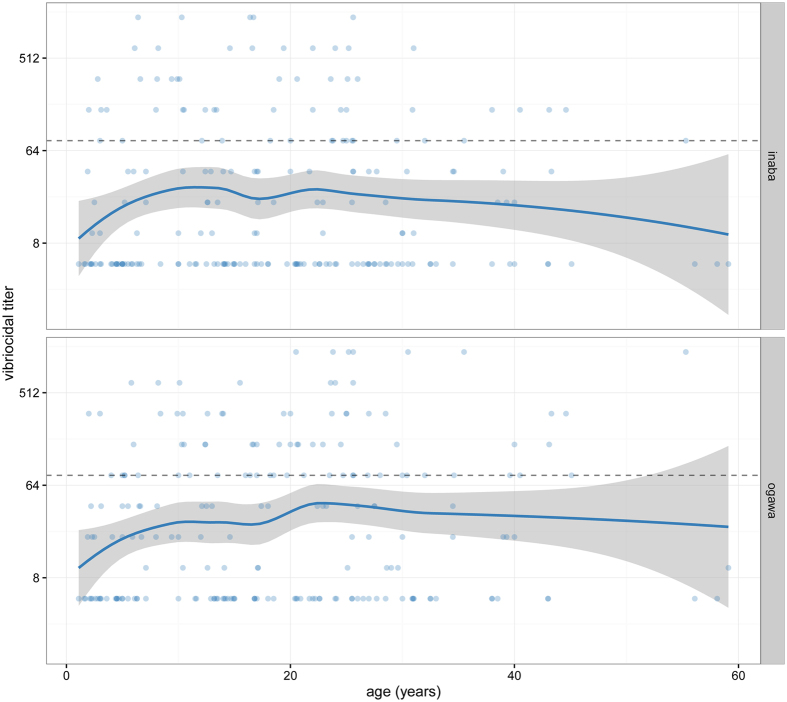
Baseline Inaba and Ogawa vibriocidal titers (y-axis, log2 transformed) by age (x-axis). Dots represent individual data points, with blue line representing smoothed (lowess) model with 95% confidence intervals in grey.

Baseline log_2_ vibriocidal titers to O1 Inaba and Ogawa ranged from 5 to 1280 in study participants. Geometric mean titers (GMT) in study participants to O1 Inaba were 21.6 (95% CI 17.1–27.4) and that to Ogawa were 28.5 (95% CI 22.4–36.2). Over one in five participants had baseline titers >80 to Inaba (21%) and Ogawa (29%). Baseline vibriocidal GMTs to both O1 serotypes generally increased with age, with older children having 2- to 3-fold higher baseline titers than younger children (Fig. 2).

Since our sample was not representative of the age-distribution of the population, we used data on the PoC age-distribution from the month of the campaign to estimate the age-standardized baseline GMTs to O1 Inaba and Ogawa. The age-standardized baseline GMTs were 26.7 (95% CI 20.9–34.0) to Ogawa and 20.6 (95% CI 16.2–26.3) to Inaba.

### Vibriocidal Response to OCV

**Figure 3:**
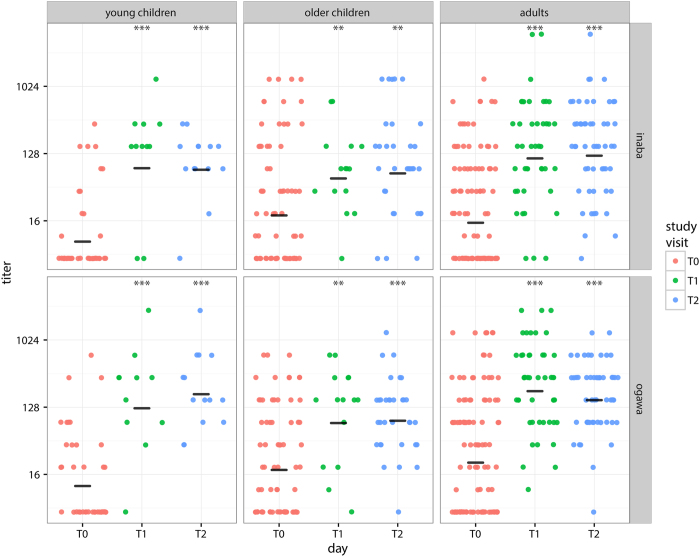
Vibriocidal titer by study visit (color), age-group (columns) and serotype (rows). Geometric mean titer (GMT) shown with black horizontal line in each. Asterisks represent p-values from tests comparing the GMT from baseline (T0) to either T1 or T2, two asterisks (**) represents p ≤ 0.01 and three asterisks (***) represents p ≤ 0.001.

Vibriocidal GMTs to Inaba and Ogawa serotypes increased significantly on day 21 (T1) and day 35 (T2) compared to baseline (T0) across all age groups (Fig. 3). Inaba vibriocidal GMTs increased significantly from 11 (95% CI 7–16) at baseline to 124 (95% CI 40–391) at T1 and 93 (95% CI 49–175) at T2 in young children; 30 (95% CI 19–48) at baseline to 73 (95% CI 36–148) and 95 (95% CI 54–166) in older children; and 22 (95% CI 16–31) at baseline to 165 (95% CI 99–274) and 152 (95% CI 102–226) in adults. Ogawa vibriocidal GMTs increased significantly from 15 (95% CI 9–25) to 170 (95% CI 57–507) and 215 (95% CI 109–425) in young children, 28 (95% CI 19–41) to 106 (95% CI 46–242) and 99 (95% CI 66–148) in older children and 36 (95% CI 25–52) to 246 (95% CI 164–369) and 182 (95% CI 135–246) in adults at T1 and T2, respectively (Fig. 3). GMTs at the two post-vaccination visits (to both serotypes) did not differ significantly from one another across the 3 age groups. While not statistically significant, on average, T1 vibriocidal GMTs were slightly higher than T2 (Fig. 3).

**Figure 4:**
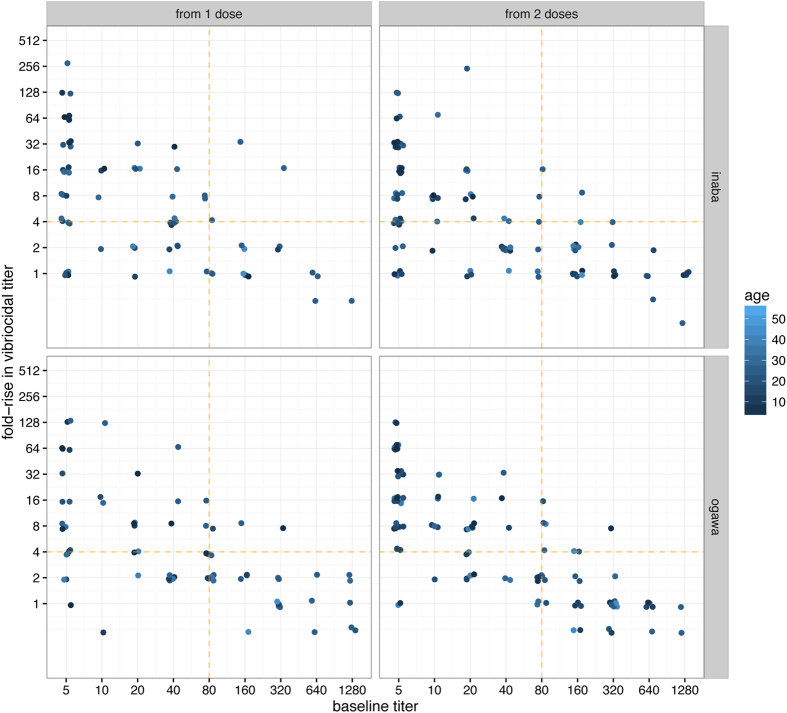
Vibriocidal responses from one and two-doses of OCV by baseline titer (x-axis), age (color) and serotype (rows). Horizontal dashed orange line represents 4 fold rise threshold; Vertical dashed line marks baseline titer 80.

Those with baseline titers ≤80 had a median (IQR) fold-rise to Inaba of 32 (10–64) in young children, 4 (2–16) in older children and 8 (2–16) in adults at T1 (maximum fold rise of 512 was in an adult at T1). Similar results were seen for Ogawa [8 (4–40), 4 (2–12), 4 (2–16) fold-rises]. Those with higher baseline titers (>80) to Inaba had lower fold-changes with a median (IQR) fold rise of 0.5 (0.03–1.0), 0.56 (0.05–1.75) and 1.5 (0.8–5.5) in young, old children and adults, respectively. Similar results were seen for Ogawa 1.5 (1–2) and 1 (0.8–2) in older children and adults (Fig. 4). In children 2–17 years old with baseline vibriocidal titers ≤80, age was associated with a significantly lower fold increases in vibriocidal response to serotype Inaba (n = 20, Spearman r = −0.58, p = 0.007) but not Ogawa (n = 19, r = −0.22; p = 0.36).

We estimated the proportion of individuals without evidence of recent exposure (baseline vibriocidal titer ≤80) who had a ≥4-fold rise in vibriocidal titer compared to baseline (seroconversion). The proportion of participants who seroconverted at T1 and T2 were similar for both serotypes. Overall, 75% (43/57) and 70% (52/74) seroconverted on T1 and T2 to Inaba and 69% (35/51) and 73% (54/74) to Ogawa. Seroconversion was more prominent in individuals with baseline titers ≤80, which was in turn inversely associated with age (Fig. 4). Young children had lower baseline titers (generally ≤40) and seroconverted more frequently, compared to adults and older children (Table 2).

We did not find significant (linear) associations between self-reported diarrhea, breastfeeding, and nutritional status on seroconversion in children under 5 years old, but analyses were limited by small sample size (Supplementary Table 1). Neither baseline nor post-vaccination vibriocidal responses differed significantly by previous OCV immunization (Supplementary Figure 1).

### OSP responses

Baseline IgM and IgA, but, not IgG, was significantly different between age groups. Baseline IgM concentrations to both Inaba and Ogawa were significantly higher in older children compared to younger children (p = 0.002 and p = 0.0004, for Inaba and Ogawa respectively) and adults (p < 0.0001 and p = 0.0008, for Inaba and Ogawa respectively). Baseline IgA concentrations to the both serotypes were significantly higher in adults compared to younger children (p = 0.007, p < 0.0001).

Significant increases in OSP IgG responses against Inaba and Ogawa were seen at T2 in all age groups compared to baseline, while significant increases in OSP IgM and OSP IgA concentrations were seen only in adults post-vaccination (Table 3). We did not find significant differences in OSP-specific IgG, IgM and IgA antibody responses between age groups, with the exception of higher Inaba IgA concentrations in adults (p = 0.011) compared to older children at T2. Similar to vibriocidal results, OSP endpoints were not significantly different at baseline and post-vaccination regardless of 2014 OCV history (Supplementary Figures 2–4).

## Discussion

We determined the immunological responses to Shanchol in a subset of South Sudanese IDPs immunized during a 2015 campaign. Our results suggest that, in this population, OCV was highly immunogenic, with no detectable differences between age groups. In addition, we found that vibriocidal and OSP-specific antibody responses to a single dose were similar to that of two doses provided approximately three weeks apart.

These findings suggest that in a population where cholera occurs regularly, like Juba, the optimal dosing schedule may be different than the currently licensed regimen, and that a single-dose may provide at least short-term protection against cholera. A recent study in a hyper-endemic setting, Dhaka, Bangladesh, estimated that a single OCV dose conferred 40% short term (6-month) protection against all medically attended cholera, and 63% against severe cholera, although the estimate in children under 5 was 16% and not significantly different than the null^27^. Similarly, another study evaluating Inaba and Ogawa specific IgA antibody secreting cells (ASC’s) in Haitian volunteers reported increases in these cell populations after the first dose but not the second^28^. Providing a second OCV dose is often challenging and increases the efforts and costs associated with vaccination campaigns, particularly in complex settings like South Sudan^23^. If a single dose can confer moderate protection on the timescale of typical cholera epidemics it could make OCV an even more cost-effective tool in emergency settings^29^.

Young children are disproportionately affected by cholera, are often at higher risk of infection (in endemic settings) and have poor disease-related outcomes^30^,^31^,^32^,^33^. Despite this, OCV may provide less protection in children under 5 years old^17^,^32^. We had hypothesized that younger children are more likely to be immunologically naïve and mount a smaller immune response to the vaccine. Despite their lower baseline titers, they were able to achieve similar vibriocidal and OSP-specific antibody responses as older individuals and adults, even after a single dose of vaccine, suggesting benefits of immunization in young children. In addition, this suggests that the vaccine may have provided immunological boosting to already primed individuals (older children and adults). However, in contrast to findings in Haiti^15^, a higher proportion of young children seroconverted compared to adults in this study and this was associated with lower baseline titers. Future vaccination strategies, including the number of doses to use, could be informed by characterizing the immunological landscape of specific populations, particularly in areas with sporadic outbreaks.

While the goal of this study was to evaluate the immunologic response to OCV, the baseline distribution of titers may reveal important insights into the historical epidemiology of cholera within the area. We found vibriocidal titers to increase with age, consistent with higher probability of historical exposure to the pathogen. Previous evidence that vibriocidal titers decay towards baseline within a year after natural infection suggest that those with high baseline titers in this study are likely a result of recent exposure to cholera^34^. The distribution of vibriocidal antibody titers, combined with models of antibody kinetics could provide clues to the intensity of historical transmission and incidence in different populations. More work is needed to help translate cross-sectional population-level distributions of cholera-specific antibodies into measures useful for cholera surveillance and to guide cholera control interventions.

Our study has several limitations. First, this is a non-randomized immunogenicity study. The study was initiated at the start of an outbreak using a convenience sample of individuals taking part in the OCV campaign, who may not have been fully, we had fewer 1–5 year olds than expected (15% observed vs. 21% expected) with 5–18 year olds over represented (37% observed vs. 28% expected). In addition, it is possible that these individuals could have been healthier and more pro-active than the general camp population. The higher percentage of people with a self-reported history of 2014 OCV immunization in this study (44%) compared to estimated overall percentage in the camp (17%) suggests this may have been true. In addition, this study was not placebo controlled, nor was there a control group so natural exposures to cholera between doses could have impacted our results. However, cholera incidence within the camp was extremely low at the time and it is unlikely that more than 1–2 individuals could have randomly been exposed during the study period. Next, due to logistical constraints, we were able to measure vibriocidal titers and OSP-specific responses only on median days 21 and 35 post-vaccination. Antibody secreting cells typically peak by day 4 in response to cholera toxoid and decline by day 7 in those re-immunized 6 months to 4 years later with OCV (Dukoral)^35^, although, post-vaccination kinetics of serum OSP and vibriocidal response is currently unknown. Since 44% of the volunteers had a self-reported history of 2014 OCV immunization, it is possible that these responses peaked earlier than our sampling, though more studies are needed to understand both vibriocidal and OSP kinetics in detail. Next, since majority of the people refused to provide follow up blood samples, there is a possibility of bias towards those with most afraid or at risk of cholera (and therefore previous exposure or vaccination). Lastly, we enrolled only 37 young children in this study of which only 11 and 15 provided blood after the first and second dose. This small sample size limited inferences related to this group including analysis of risk factors for non-seroconversion like nutritional status, history of cholera and OCV.

In conclusion, we found that Shanchol was immunogenic in this cohort of South Sudanese IDPs as confirmed by increases in vibriocidal and OSP-specific antibody responses. The lack of differences in the vaccine responses between children and adults are reassuring, as immunologically naive young children may have the most to benefit from vaccination, although our study was not powered to detect differences. In addition, our findings that one dose of OCV elicited a similar immune response as two doses provides hope that a single-dose strategy may provide at least short-term protection in some populations^27^. Though OCV is a feasible and increasingly used public health tool, more work is needed to understand differences in protection from OCV across sub-populations and how alternative dosing regimens, tailored to the background immunologic profile of the population, may enhance protection and operational feasibility.

## Additional Information

**How to cite this article**: Iyer, A. S. *et al.* Immune Responses to an Oral Cholera Vaccine in Internally Displaced Persons in South Sudan. *Sci. Rep.* **6**, 35742; doi: 10.1038/srep35742 (2016).

## Supplementary Material

Supplementary Information

## Figures and Tables

**Table 1:** Demographics of study participants.

Group	Younger Children (1–5)		Older Children (6–17)		Adults > 18	
Baseline Vibriocidal Titer	≤80	>80	≤80	>80	≤80	>80
Number	33	4	49	18	80	21
Mean age (S.D.)	3.7 (1.4)	2.8 (0.6)	12.8 (3.4)	10.7 (3.4)	29.5 (9.1)	26.7 (8.4)
Male	16/33 (48%)	1/4 (25%)	24/49 (49%)	6/18 (33%)	25/80 (31%)	2/21 (10%)
Reported at least 1-day with diarrhea in the week before dose 1 of OCV	10/33 (30%)	0/4 (0%)	5/49 (10%)	5/18 (28%)	8/80 (10%)	1/21 (5%)
Self reported cholera history before dose 1 of OCV	1/33 (3%)	0/4 (0%)	1/49 (2.0%)	0/18 (0%)	3/80 (4%)	2/21 (10%)
Self reported receipt of OCV in 2014	11/33 (33%)	1/3 (33%)	25/49 (51%)	13/18 (72%)	33/80 (41%)	6/21 (29%)
Doses in 2014 (1/2/3)	7/4/0	0/1/0	14/10/1	7/6/0	11/20/2	3/3/0

**Table 2:** Vibriocidal seroconversion (number and proportion) after doses 1(T1) and 2(T2) in participants with or without evidence of recent cholera exposure.

Vibriocidal Seroconversion	Young Children				Older Children				Adults			
	Baseline titer ≤ 80		Baseline titer > 80		Baseline titer ≤ 80		Baseline titer > 80		Baseline titer ≤ 80		Baseline titer > 80	
	Dose 1	Dose 2	Dose 1	Dose 2	Dose 1	Dose 2	Dose 1	Dose 2	Dose 1	Dose 2	Dose 1	Dose 2
Inaba	9/10 (90%)	12/13 (92%)	0/2 (0%)	0/2 (0%)	8/11 (73%)	12/22 (56%)	0/4 (0%)	0/10 (0%)	26/36 (72%)	28/39 (72%)	2/10 (20%)	3/15 (20%)
Ogawa	8/10 (80%)	10/12 (83%)	1/1 (100%)	1/2 (50%)	8/13 (62%)	18/25 (72%)	0/2 (0%)	0/8 (0%)	19/28 (68%)	26/37 (70%)	1/18 (5%)	2/17 (12%)

**Table 3:** OSP-specific IgG, IgM and IgA responses after dose 1(T1) and 2(T2). Asterisks indicate significant increases in post-vaccination levels compared to baseline.

Parameters	Young Children Geometric Mean (95% C.I.)			Older Children Geometric Mean (95% C.I.)			Adults Geometric Mean (95% C.I.)		
	Baseline	Dose 1	Dose 2	Baseline	Dose 1	Dose 2	Baseline	Dose 1	Dose 2
**IgG**									
Inaba	44 (37–54)	78 (30–206)	134 (61–294)**	41 (36–46)	54 (38–77)	87 (62–120)****	41 (36–47)	54 (39–75)	68 (51–92)**
Ogawa	25 (20–31)	46 (20–108)	42 (21–85)	26 (23–30)	43 (29–65)*	44 (33–59)**	30 (26–34)	56 (40–77)**	51 (38–69)**
**IgM**									
Inaba	60 (50–73)	80 (46–140)	78 (53–115)	84 (73–96)	74 (55–100)	92 (78–108)	58 (51–65)	81 (65–100)**	82 (68–100)***
Ogawa	42 (35–49)	55 (33–94)	55 (42–71)*	61 (53–69)	55 (41–72)	70 (57–85)	44 (38–52)	65 (52–82) **	52 (43–62)
**IgA**									
Inaba	23 (19–29)	34 (20–59)	38 (24–62)	28 (24–34)	38 (24–62)	37 (27–50)	34 (29–40)	63 (48–82)***	63 (48–82)***
Ogawa	9 (7–12)	12 (7–22)	12 (7–21)	14 (12–16)	19 (12–30)	14 (12–18)	17 (14–20)	24 (18–31)	23 (17–30)

*p ≤ 0.05, **p ≤ 0.01, ***p ≤ 0.001, ****p ≤ 0.0001.

## References

[b1] World Health Organization (WHO). Weekly epidemiological record, Cholera, 2014. http://www.who.int/wer/2015/wer9040.pdf?ua=1 10/02/ 2015. Accessed on 01/05/2016.

[b2] World Health Organization, Government of the Republic of South Sudan. Situation Report #89 on Cholera in South Sudan as at 23:59 Hours, 12–18 October 2015 http://reliefweb.int/report/south-sudan/situation-report-89-cholera-south-sudan-2359-hours-12-18-october-2015 12-18 October 2015. Accessed on 03/04/2016.

[b3] World Health Organization, Republic of South Sudan http://www.who.int/hac/crises/ssd/south_sudan_ewarn_21december2014.pdf 15-21 December 2014 Accessed on 04/21/2016.

[b4] Harris, *et al.* Immunologic responses to Vibrio cholerae in patients co-infected with intestinal parasites in Bangladesh. PLoS Neglected Tropical Diseases 3, e403, doi: 10.1371/journal.pntd.0000403 (2009). 19333369 PMC2657204

[b5] Bhattacharya S. K. *et al.* 5 year efficacy of a bivalent killed whole-cell oral cholera vaccine in Kolkata, India: a cluster-randomised, double-blind, placebo-controlled trial. Lancet Infect Dis 13, 1050–1056, doi: 10.1016/S1473-3099(13)70273-1 (2013). 24140390

[b6] Grassly N. C. *et al.* New strategies for the elimination of polio from India. Science 314, 1150–1153, doi: 10.1126/science.1130388 (2006). 17110580

[b7] Jiang V., Jiang B., Tate J., Parashar U. D. & Patel M. M. Performance of rotavirus vaccines in developed and developing countries. Hum Vaccin 6, 532–542 (2010). 20622508 10.4161/hv.6.7.11278 PMC3322519

[b8] Kotloff K. L. *et al.* Safety and immunogenicity in North Americans of a single dose of live oral cholera vaccine CVD 103-HgR: results of a randomized, placebo-controlled, double-blind crossover trial. Infect Immun 60, 4430–4432 (1992). 1398956 10.1128/iai.60.10.4430-4432.1992 PMC257485

[b9] Suharyono *et al.* Safety and immunogenicity of single-dose live oral cholera vaccine CVD 103-HgR in 5-9-year-old Indonesian children. Lancet 340, 689–694 (1992). 1355798 10.1016/0140-6736(92)92231-4

[b10] Martin S. *et al.* Post-licensure deployment of oral cholera vaccines: a systematic review. Bull World Health Organ 92, 881–893, doi: 10.2471/BLT.14.139949 (2014). 25552772 PMC4264394

[b11] Ali M. *et al.* Herd protection by a bivalent killed whole-cell oral cholera vaccine in the slums of Kolkata, India. Clin Infect Dis 56, 1123–1131, doi: 10.1093/cid/cit009 (2013). 23362293

[b12] Ivers L. C. *et al.* Use of oral cholera vaccine in Haiti: a rural demonstration project. Am J Trop Med Hyg 89, 617–624, doi: 10.4269/ajtmh.13-0183 (2013). 24106187 PMC3795090

[b13] Luquero F. J. *et al.* Use of Vibrio cholerae vaccine in an outbreak in Guinea. N Engl J Med 370, 2111–2120, doi: 10.1056/NEJMoa1312680 (2014). 24869721

[b14] Khatib A. M. *et al.* Effectiveness of an oral cholera vaccine in Zanzibar: findings from a mass vaccination campaign and observational cohort study. Lancet Infect Dis 12, 837–844, doi: 10.1016/S1473-3099(12)70196-2 (2012). 22954655

[b15] Charles R. C. *et al.* Immunogenicity of a killed bivalent (O1 and O139) whole cell oral cholera vaccine, Shanchol, in Haiti. PLoS neglected tropical diseases 8, e2828, doi: 10.1371/journal.pntd.0002828 (2014). 24786645 PMC4006712

[b16] Leung D. T. *et al.* Immune responses to the O-specific polysaccharide antigen in children who received a killed oral cholera vaccine compared to responses following natural cholera infection in Bangladesh. Clin Vaccine Immunol 20, 780–788, doi: 10.1128/CVI.00035-13 (2013). 23515016 PMC3675980

[b17] Sur D. *et al.* Efficacy of a low-cost, inactivated whole-cell oral cholera vaccine: results from 3 years of follow-up of a randomized, controlled trial. PLoS neglected tropical diseases 5, e1289, doi: 10.1371/journal.pntd.0001289 (2011). 22028938 PMC3196468

[b18] Sur D. *et al.* Efficacy and safety of a modified killed-whole-cell oral cholera vaccine in India: an interim analysis of a cluster-randomised, double-blind, placebo-controlled trial. Lancet 374, 1694–1702, doi: 10.1016/S0140-6736(09)61297-6 (2009). 19819004

[b19] Desai S. N. *et al.* A Randomized, Placebo-Controlled Trial Evaluating Safety and Immunogenicity of the Killed, Bivalent, Whole-Cell Oral Cholera Vaccine in Ethiopia. The American journal of tropical medicine and hygiene 93, 527–533, doi: 10.4269/ajtmh.14-0683 (2015). 26078323 PMC4559691

[b20] Chen W. H. *et al.* Single-dose Live Oral Cholera Vaccine CVD 103-HgR Protects Against Human Experimental Infection With Vibrio cholerae O1 El Tor. Clin Infect Dis 62, 1329–1335, doi: 10.1093/cid/ciw145 (2016). 27001804 PMC4872293

[b21] Losonsky G. A. *et al.* Factors influencing secondary vibriocidal immune responses: relevance for understanding immunity to cholera. Infect Immun 64, 10–15 (1996). 8557325 10.1128/iai.64.1.10-15.1996 PMC173720

[b22] Johnson R. A. *et al.* Comparison of immune responses to the O-specific polysaccharide and lipopolysaccharide of Vibrio cholerae O1 in Bangladeshi adult patients with cholera. Clin Vaccine Immunol 19, 1712–1721, doi: 10.1128/CVI.00321-12 (2012). 22993410 PMC3491541

[b23] Abubakar A. *et al.* The First Use of the Global Oral Cholera Vaccine Emergency Stockpile: Lessons from South Sudan. PLoS Med 12, e1001901, doi: 10.1371/journal.pmed.1001901 (2015). 26576044 PMC4648513

[b24] Azman A. S. *et al.* Population Level Effect of Cholera Vaccine on Displaced Populations, South Sudan. CDC. 22, 6—June 2016. doi: 10.3201/eid2206.151592 (2014). PMC4880069 27192187

[b25] International Organization for Migration, South Sudan. IOM Supports Cholera Response in Juba http://southsudan.iom.int/media-and-reports/press-release/iom-supports-cholera-response-juba. 25 September 2015. Accessed on 04/20/2016.

[b26] Sayeed M. A. *et al.* A Cholera Conjugate Vaccine Containing O-specific Polysaccharide (OSP) of V. cholerae O1 Inaba and Recombinant Fragment of Tetanus Toxin Heavy Chain (OSP:rTTHc) Induces Serum. Memory and Lamina Proprial Responses against OSP and Is Protective in Mice. PLoS Neglected Tropical Diseases 9, e0003881, doi: 10.1371/journal.pntd.0003881 (2015). 26154421 PMC4495926

[b27] Qadri F. *et al.* Efficacy of a Single-Dose, Inactivated Oral Cholera Vaccine in Bangladesh. N Engl J Med 374, 1723–1732, doi: 10.1056/NEJMoa1510330 (2016). 27144848

[b28] Matias W. R. *et al.* Antibody Secreting Cell Responses following Vaccination with Bivalent Oral Cholera Vaccine among Haitian Adults. PLoS neglected tropical diseases 10, e0004753, doi: 10.1371/journal.pntd.0004753 (2016). 27308825 PMC4911095

[b29] Azman A. S. *et al.* The Impact of a One-Dose versus Two-Dose Oral Cholera Vaccine Regimen in Outbreak Settings: A Modeling Study. PLoS Med 12, e1001867, doi: 10.1371/journal.pmed.1001867 (2015). 26305226 PMC4549326

[b30] Deen J. L. *et al.* The high burden of cholera in children: comparison of incidence from endemic areas in Asia and Africa. PLoS neglected tropical diseases 2, e173, doi: 10.1371/journal.pntd.0000173 (2008). 18299707 PMC2254203

[b31] Leung D. T., Chowdhury F., Calderwood S. B., Qadri F. & Ryan E. T. Immune responses to cholera in children. Expert Rev Anti Infect Ther 10, 435–444, doi: 10.1586/eri.12.23 (2012). 22512753 PMC3378723

[b32] Sinclair D., Abba K., Zaman K., Qadri F. & Graves P. M. Oral vaccines for preventing cholera. *Cochrane Database Syst Rev*, CD008603 , doi: 10.1002/14651858.CD008603.pub2 (2011). PMC6532691 21412922

[b33] Masuet Aumatell C., Ramon Torrell J. M. & Zuckerman J. N. Review of oral cholera vaccines: efficacy in young children. Infect Drug Resist 4, 155–160, doi: 10.2147/IDR.S10339 (2011). 22114507 PMC3215343

[b34] Harris A. M. *et al.* Antigen-specific memory B-cell responses to Vibrio cholerae O1 infection in Bangladesh. Infect Immun 77, 3850–3856, doi: 10.1128/IAI.00369-09 (2009). 19528207 PMC2738048

[b35] Leach S., Lundgren A. & Svennerholm A. M. Different kinetics of circulating antibody-secreting cell responses after primary and booster oral immunizations: a tool for assessing immunological memory. Vaccine 31, 3035–3038, doi: 10.1016/j.vaccine.2013.04.066 (2013). 23664997

