# Supplementary material for: Immune Responses to an Oral Cholera Vaccine in Internally Displaced Persons in South Sudan
Source: Sci Rep. 2016 Oct 24;6:35742. doi: 10.1038/srep35742 (PMC5075787; doi:10.1038/srep35742)

Immune Responses to an Oral Cholera Vaccine in Internally Displaced Persons in South Sudan.

Anita S. Iyer[1#], Malika Bouhenia[2#], John Rumunu[3], Abdinasir Abubakar[2], Randon J. Gruninger[1], Jane Pita[2], Richard Lako[3], Lul Deng[3], Joseph F. Wamala[2], Edward T. Ryan[4], Stephen Martin[5], Dominique Legros[5], Justin Lessler[6], David Sack[7], Francisco J. Luquero[7,8], Daniel T. Leung[1,9$], Andrew S. Azman[6$*]

Captions for Supplementary Figures and Tables:

Supplementary Table 1: Muac Z, WAZ and HAZ scores in young children by seroconversion.

[Note: In case of children 5 years old, we calculated their anthropometrics as 60 month-olds]

Parameters	Vibriocidal Seroconverters	Vibriocidal Non-Seroconverters
Breastfed at T0	4/12 (33%)	1/4 (25%)
MUAC score [Median (25%, 75% IQR)]	[-0.77 (-1.1,-0.69)]	[-0.02 (-1.4, 0.06)]
WAZ score [Median (25%, 75% IQR)]	[-0.69 (-1.2, 0.14)]	[-0.69 (-1.0,0.05)]
HAZ score [Median (25%, 75% IQR)]	[-0.51 (-1.4,0.17)]	[-0.03 (-1.7,2.0)]

Supplementary Figure 1: Vibriocidal titer by study visit (x-axis), age-group (columns), serotype (rows), stratified by OCV history. Geometric mean titer (GMT) shown with horizontal line in each.

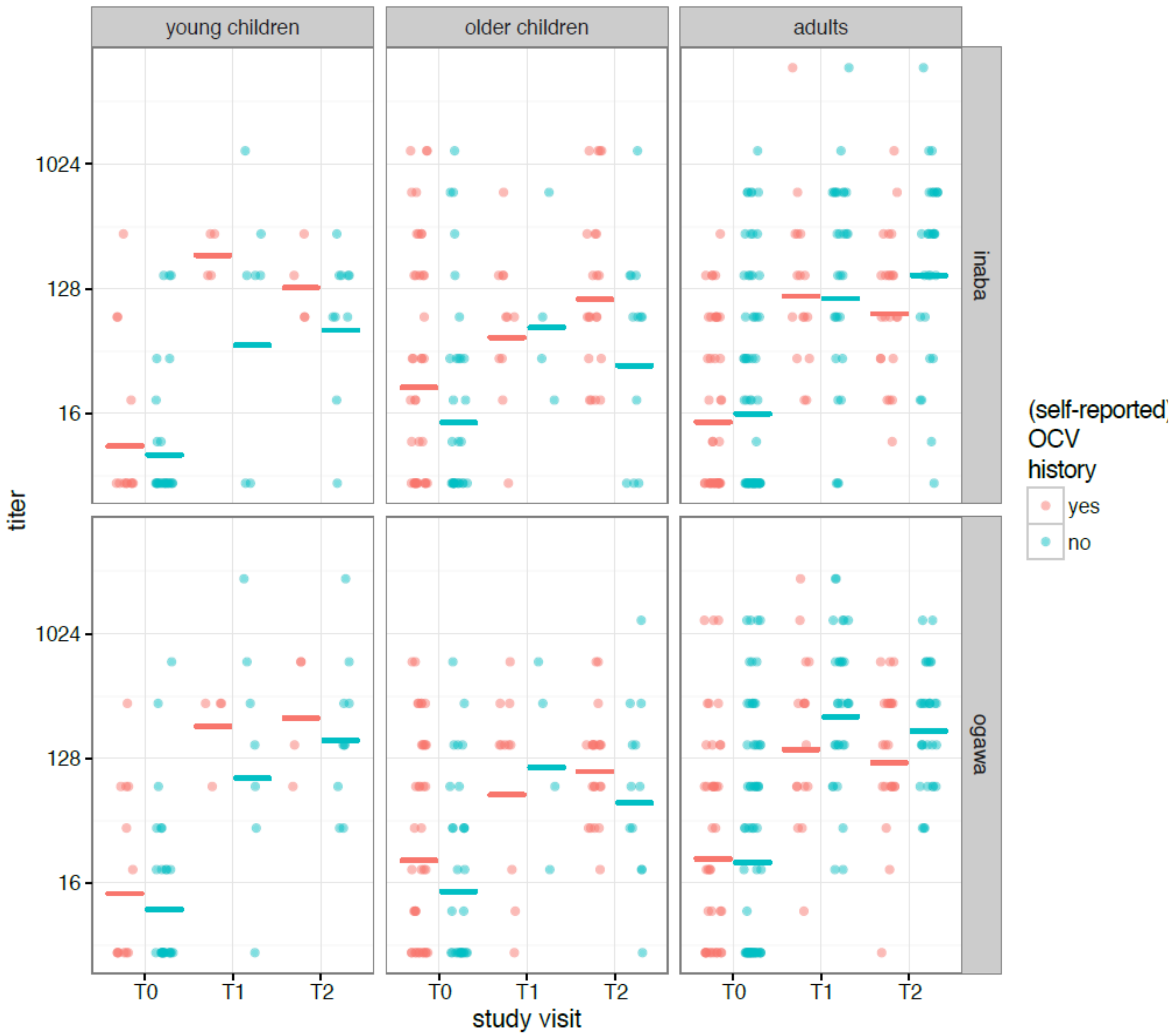

Supplementary Figure 2: OSP IgA response by study visit (x-axis), age-group (columns), serotype (rows), stratified by OCV history. Geometric mean titer (GMT) shown with horizontal line in each.

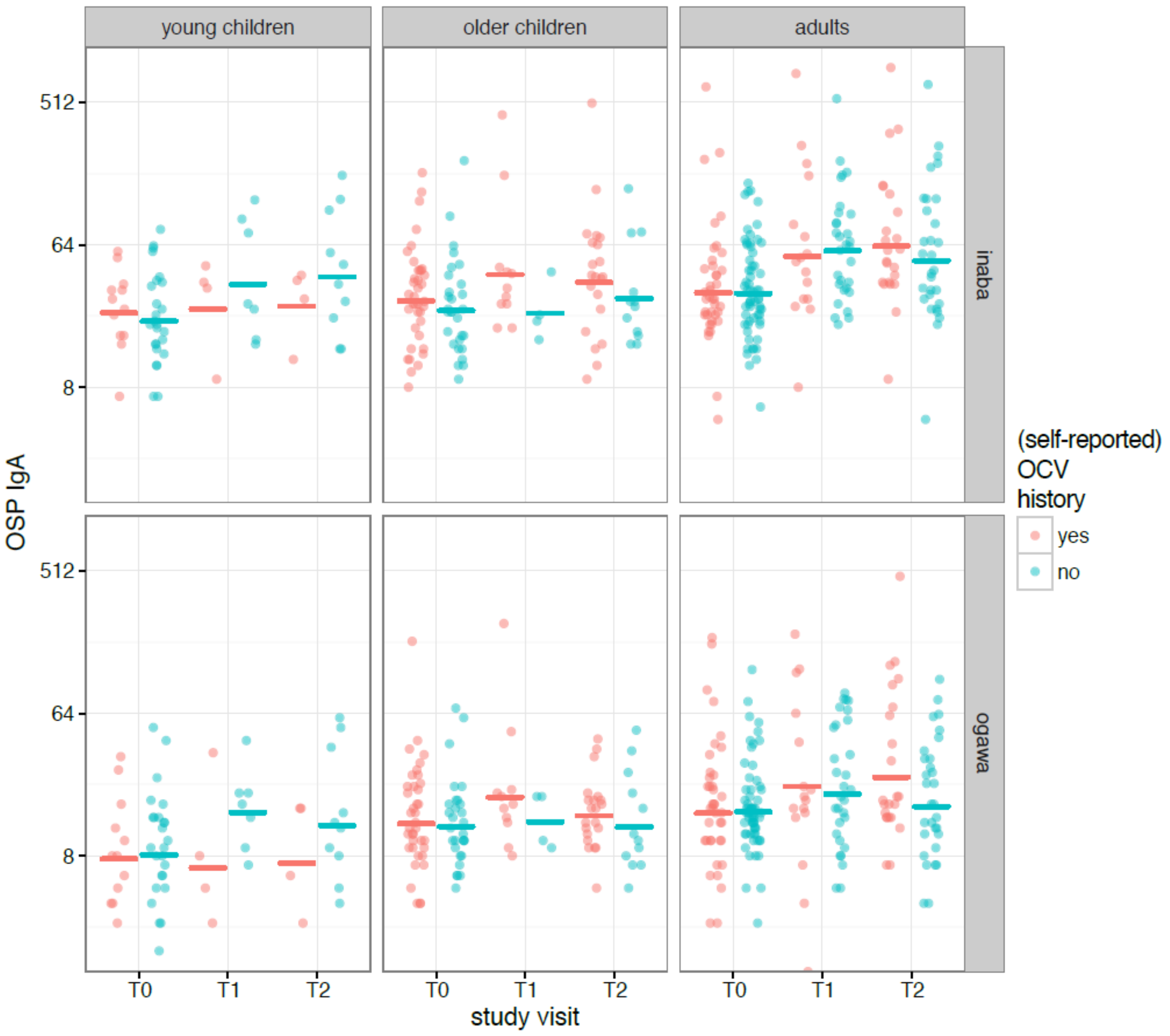

Supplementary Figure 3: OSP IgM response by study visit (x-axis), age-group (columns), serotype (rows), stratified by OCV history. Geometric mean titer (GMT) shown with horizontal line in each.

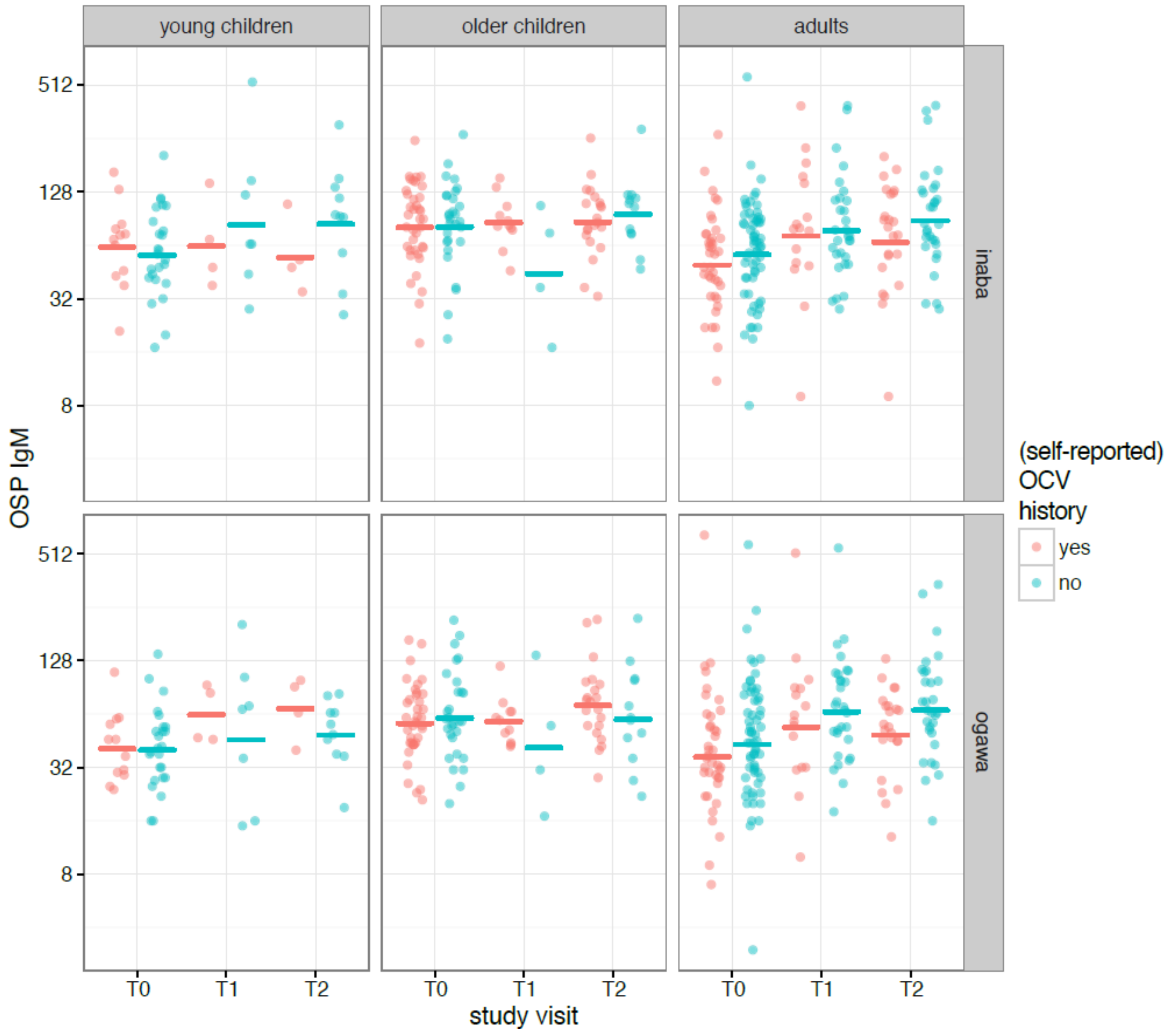

Supplementary Figure 4: OSP IgG response by study visit (x-axis), age-group (columns), serotype (rows), stratified by OCV history. Geometric mean titer (GMT) shown with horizontal line in each.

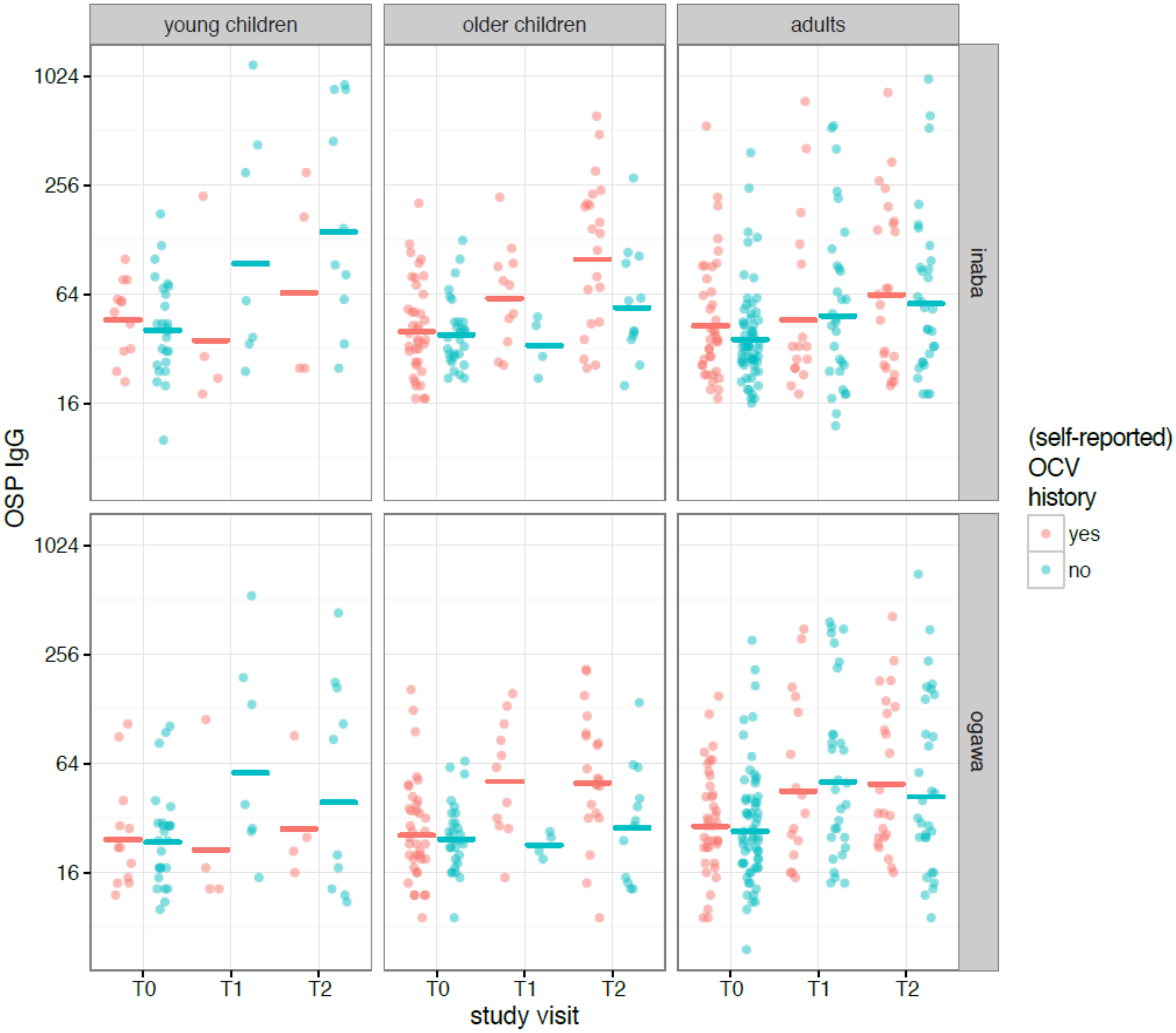

Supplement: Supplementary Information [file srep35742-s1.pdf]
